# A Fish-Hook Removed from the Œsophagus without an Operation

**Published:** 1845-03

**Authors:** 


### Collectanea.

*A Fish-Hook removed from the Œsophagus without an Operation.*—In the summer of 1837, Mrs. * * * was enjoying her usual *siesta,* in the afternoon of a warm day, on a pallet spread upon the floor in a cool part of the house: and while she was lying on her back sleeping pleasantly, no doubt dreaming of past pleasures, her grandson, a little urchin of three or four summers, was playing about the house with a fishing tackle complete, pole, line and hook; who, when he discovered the old lady with her mouth widely distended, thought it was a fine opportunity to "catch a fish." Accordingly, in order to effect his purpose, he cautiously deposited the "barbed hook," (I believe there was no *bait* on it,) into his granddame's open mouth. The titilation caused her to awake suddenly, and as her mouth was dry from exposure, she closed it, and swallowed the hook two or three inches below the uvula. So soon as she discovered her situation, the whole family was assembled by her calls and cries of distress, *except little Charley,* who had dropped his pole in a panic, and, in provincial phrase, *mizzled.*

Some gentle efforts were essayed to remove the hook, both by the patient and some of the family, but being apprehensive of fixing the barb in the throat, they ceased all efforts, and despatched a messenger for Dr. E. Leroy Antony, who resided in the neighborhood. When he arrived, and found that the hook was not fastened into the flesh, his fertile brain suggested a plan by which it could be removed safely, easily, and *without an operation.*

His plan was, to cut off the line within a foot or two of the mouth of the patient; then to drill a hole through a rifle bullet and drop it over the line, down on the hook. In order to fix the bullet on the point of the hook, and maintain it firmly in that position, a reed was procured, the joints punched out, and then passed down over the line, and pressed firmly over the bullet. In this manner the hook, bullet and reed, were all withdrawn at once, very easily, without any injury to the œsophagus or fauces.

This all seems so simple, like Columbus' egg, that the reader may think he would have done just the same thing. But the influence of education and of common practice, and the desire to perform surgical operations and acquire some celebrity, all conspire to keep us in the same beaten track; and the majority of minds, when started and trained in a certain way, seldom, if ever, alter their course.

It is matter of rejoicing too, that the knife is less used now, than it was some years since, when surgeons seemed to vie with each other who could cut the largest gashes and the most of them.

The above case occurred in Barnwell District, S. C., and, as stated above, was treated by Dr. Edwin Leroy Antony, son of Dr. Milton Antony, of Augusta, Georgia. Both were carried off, in 1839, by the epidemic yellow fever of that city; and society and the profession lost two bright ornaments, Dr. E. L. Antony seemed to be formed a physician by nature; he possessed talents of a rare order, and an intuitive knowledge of nature and disease. As to his father, Dr. M. Antony, no eulogy can add to his fame; his name is rendered immortal by the Medical College of Georgia.—*New Orleans Med. Jour.*

